# LGBTQ+ health research guides: a multi-institutional analysis of usage patterns and user information needs

**DOI:** 10.5195/jmla.2023.1661

**Published:** 2023-10-02

**Authors:** Gregg A. Stevens, Martin Morris, Robin M. N. Parker, Francisco J. Fajardo, Erica R. Brody, Katie McLean

**Affiliations:** 1 Gregg.Stevens1@umassmed.edu, Manager, Library Education and Clinical Services, University of Massachusetts Chan Medical School, Worcester, MA, United States.; 2 martin.morris@mcgill.ca, Associate Librarian, Schulich Library of Physical Sciences, Life Sciences and Engineering, McGill University, Montreal, QC, Canada.; 3 robin.parker@dal.ca, Evidence Synthesis Librarian, W. K. Kellogg Health Sciences Library, Dalhousie University, Halifax, NS, Canada.; 4 ffajardo@fiu.edu, Assistant Director, Public and Information Services, Herbert Wertheim College of Medicine Medical Library, Florida International University, Miami, FL, United States.; 5 ebrody@vcu.edu, Research and Education Librarian, Health Sciences Library, Virginia Commonwealth University, Richmond, VA, United States.; 6 katie.mclean@nshealth.ca, Librarian Educator, Nova Scotia Health Authority, Halifax, NS, Canada.

**Keywords:** LGBTQ+, health information, research guides, health disparities, health sciences libraries, consumer health, outreach, libguides

## Abstract

**Objective:**

LGBTQ+ health research guides can strengthen the LGBTQ+ community through connecting people to quality health services and information, and previous studies have recommended that health sciences libraries create and maintain these guides. Little evidence exists, though, on how these guides are used and how well they meet the needs of LGBTQ+ users. Using retrospective data retrieved from multiple LGBTQ+ health research guides, we examined the categories of LGBTQ+ health information most used, as well as how often guides were accessed. Based on these results, we hope to find patterns which can lead to best practices for libraries.

**Methods:**

Five North American academic health sciences libraries contributed select usage data from their LGBTQ+ health research guides, covering a three-year period (July 2018-June 2021). Data was analyzed in two ways. Firstly, the 20 most-clicked resources from each guide were categorized through open coding, to assess if certain information resource categories were more popular among guide users, allowing for inference of user needs. A time-series analysis was also conducted for two sites, using the Classical Seasonal Decomposition by Moving Averages method, to provide deeper insights into the data.

**Results:**

Open coding data showed consumer health information resources were used more often than other health resource categories. Resources from more locally based organizations and those with provider and services information were heavily used, indicating that users may be looking for information connecting to local health services and providers. The time series analysis allowed the potential positive effect of guide promotion to be showcased in ways that would not have been clear from the raw data.

**Conclusion:**

This study shows that people are accessing LGBTQ+ consumer health information through academic library research guides, with a preference for local information. Guide usage appears to be positively driven by outreach within one's institution and to the greater community. Locating external partners may increase guide impact and provide important links to local resources and services.

## INTRODUCTION

The lesbian, gay, bisexual, transgender, queer, and plus (LGBTQ+) community has a well-documented history of challenges relating to equitable healthcare, and these challenges continue today. Some are related to receiving healthcare that is both equitable in quality and affirming in delivery [1–3]. Other challenges relate to increased risk of mental health or substance abuse issues for members of the community [4–6]. Yet other challenges are the result of disparities related to sexual or social behaviors, leading to higher incidences of diseases ranging from cancer and cardiovascular disease in lesbians and bisexual women to higher rates of human immunodeficiency virus (HIV) and other sexually transmitted infections in gay and bisexual men [7–9]. Transgender persons wishing to seek gender-affirming medical treatments related to their transitions have a unique set of needs [10,11]. As an overarching consideration, LGBTQ+ persons must confront misinformation, either through malice or ignorance, which makes the search for relevant quality information an even greater challenge [12,13]. With such challenges, there is an undeniable need to make sure that LGBTQ+ persons and their healthcare providers have access to quality health information resources.

In a highly welcome change, a steadily increasing number of medical, dental, nursing, and other health sciences programs now include LGBTQ+ health, and the provision of LGBTQ+ affirming care [14–17]. Research indicates that healthcare professionals can benefit from continuing education on LGBTQ+ healthcare competencies and health information [18]. Despite this increased focus on LGBTQ+ health in curricula and clinical practice, very little research has been conducted into how healthcare professionals and healthcare students search for information to support their LGBTQ+ patients. Fikar and Keith's seminal study into the information needs and information-seeking behavior of LGBTQ health professionals found that due to concerns that they may experience discrimination, many LGBTQ health professionals have distinct information needs, prefer to seek LGBTQ health information from a medical librarian who is also LGBTQ, and want medical libraries to create a more LGBTQ-friendly environment through the use of visible signs of support and targeted Web resources such as dedicated subject guides [19]. Morris & Roberto's follow-up study largely confirmed these results, finding that little had changed in the intervening decade [20]. They also recommended that relevant libraries have a dedicated specialist in LGBTQ health information and that a collaboration between the library and interested medical faculty could be profitable in this context. Literature in both the health sciences and in library and information science describes LGBTQ+ health information seeking and how those needs are met. As an early example of librarians working to provide LGBTQ+ health information, some librarians created print reference materials to link consumers to community resources for HIV/AIDS, providing pre-internet access to information when reliable information was not always easy to access or identify [21–24]. Recent studies have focused on health information seeking by sexual and gender minority youth and information seeking by men who have sex with men (MSM) on topics such as pre-exposure prophylaxis (PrEP), a medication that decreases the risk of HIV transmission [25,26, 27]. Other studies have focused on information seeking by transgender and gender diverse persons, in areas such as breast cancer screening and human papillomavirus vaccination [28,29]. In a library-centered study, Drake and Bielefield reported on the information needs of transgender library patrons. They determined that online resources were the preferred type of information sources by the people in their study, including when seeking physical and mental health information [30].

A small body of literature has moved away from the focus on the information seeker, whether patient or healthcare provider, to examine the role of librarians and library professionals in providing LGBTQ+ health information. Morris and Hawkins and Hawkins et al. highlighted ways open to libraries wishing to provide valuable and trustworthy health information to LGBTQ+ users. Of note, they encourage librarians to create and maintain guides, addressing both consumer health questions as well as those of healthcare providers [31,32]. Stevens et al. build on this work by mapping the landscape of health sciences librarian outreach to LGBTQ+ people through case studies, thus highlighting how the health sciences librarian can play a valuable role as an activist for LGBTQ+ health information and advocate for the health of the LGBTQ+ community through proactive information sharing and outreach [33]. A significant part of this evidence map is the use of library guides. Further work by Stevens and Fajardo assesses the scope of existing LGBTQ+ health research guides by analyzing the content of 74 such guides at academic and hospital libraries in the United States and Canada. Sadly, they also found that many major health sciences libraries were lacking such guides, missing an opportunity to provide information and outreach to their LGBTQ+ audiences [34].

While this work focused on the guides in relation to libraries and librarians, there are few studies focused specifically on research guides from the user's perspective. Ouellette conducted a qualitative study to determine how students use guides and what they like about them. The students reported that they liked the guides they were presented with but admitted they did not often use them. They felt that, if the guides were better tailored to their specific needs, then they would probably consult them more [35]. More recently, Kouame and Hendren assessed the clinical information needs of registered nurses at a nursing home through a mixed-methods study. To address the nurses' needs, the librarians created a guide, which they stated was well received by the nurses [36]. Both studies indicate that guides are valued positively by users.

Despite this work, however, little is known about the content and format that users value most in a health information guide, or whether the specific types of information needs are met through accessing a guide, ultimately driving guide usage. This work aims to address that gap in our knowledge and has two objectives. First, we wanted to assess LGBTQ+ health information guide usage generally, including their usage over time and any usage patterns that could be discerned. Secondly, based on usage patterns from LGBTQ+ health research guides, we planned to assess the types of LGBTQ+ health information of greatest interest to health sciences library users and how, if appropriate, these guides might be revised to be more relevant to user needs.

## METHODS

The authors of this study represent five libraries with LGBTQ+ health guides all of which are housed on Springshare's LibGuides platform. Libraries were selected through purposive sampling based on existing knowledge of peers who have created relevant guides and the availability of guide access data [37,38]. Two different data sets were provided by each participating library, each set covering a three-year time period from July 2018 through June 2021.

### Case Selection: Vignettes

The research guides we selected for this analysis each have a different origin and purpose. For example, one is a partnership project with a local health authority library and a public library, while another was originally created as part of a public health outreach program. This means that, while it is possible to draw some generalized conclusions about their usage, we have also found it useful to conduct customized analyses where possible. For this reason, we have chosen to present each guide in our study separately through a series of vignettes, a model previously employed in other studies [39].

### Data Collection and Analysis

For the first data set for this study, we generated data sets detailing the total number of clicks over the three-year period for each of the guide's individual resources, or “assets” as they are termed in the LibGuides platform. Springshare usage data was chosen as it was easily accessible by all participating libraries using LibGuides and could produce uniform data sets. The top 20 most used links were identified for each guide, to provide a sample size that would be large enough to be meaningful but would also eliminate the need to tabulate links with very small numbers of clicks. These links were categorized using open coding to determine basic characteristics of each resource. Twelve categories were established for coding of the resources. The total number of clicks for each resource was used to weight the coding results, yielding a total number of clicks per code. One resource could be coded for more than one category. As this was a collaborative project across institutions, Google Sheets was chosen for its simplicity and shareability. (See Supplemental Data).

Two sites, Florida International University and the Nova Scotia Health/Dalhousie University collaboration, had usage numbers high enough to facilitate deeper analyses of the data. Although we planned to conduct similar analyses for the other sites, the data for these guides were insufficient. For these two sites we generated a second data set: the monthly overall guide usage over the time period, as calculated by the total number of guide clicks for each month. This data covered the same three-year time period, comprising 36 data points. For the Florida International University guide we obtained an additional year's data (July 2021-June 2022), comprising a total of 48 data points over a 4-year period. For the Nova Scotia guide the usage numbers for two of its four subpages (“About”: an introduction to the guide and “Community”: covering consumer health information) were each high enough to permit separate analyses. Two others (“Healthcare Providers and Trainees” and “Researchers”) had usage figures too low to allow this analysis.

Using these second datasets, we conducted a time series analysis in R (version 4.2.1) via RStudio (version 2022.07.2), using the TSStudio package [40, 41, 42]. We employed the “Classical Seasonal Decomposition by Moving Averages” methodology as outlined in Krispin and Shumway & Stoffer [43, 44]. This is a common method for analyzing time series data, and through it we aimed to determine any possible underlying insights in the data which would not be visible from a simple visual examination of the raw data. These factors could include seasonal variations and the additional effect of any ad-hoc factors such as promotional campaigns and special events. The syntax for this analysis is given in Appendix 1. As this is an exploratory analysis of a small dataset, we did not conduct a power analysis. We comment on this further in the results section below.

### Vignette #1: Florida International University

The Transgender Resources Guide at Florida International University (FIU) [45] was created in July 2015 by one of the librarians (FJF) at the Herbert Wertheim College of Medicine Medical Library. The guide was created as part of a study funded by the Miami Foundation to analyze the transgender female population in South Florida and offer local resources to services for participants [46]. The transgender resources guide offered seven individual tabs with information on local health care and counseling services, community and government resources, current news/events, films, books, lists of providers in the South Florida specializing in transgender health care, and links on where to obtain PrEP.

The guide includes a provider list for caregivers specializing in transgender health within South Florida. The guide's creator contacted various nonprofit organizations and consulted with leaders to compile the list. One crucial standout in the guide contains information on where to access PrEP and Post-Exposure Prophylaxis (PEP), an infographic about the drug, and other information from the CDC.

Since its publication, the guide has been used by various community members, university faculty, and staff to supplement diversity training such as Safe Zone. For this reason, the Transgender Resources guide is updated frequently with new content for education and to assist with healthcare linkages to this population.

### Vignette #2: Nova Scotia Health/Dalhousie University

The original version of the 2SLGBTQIA+ Health Guide [47] was created by librarians (RP and KM) at Dalhousie University and Nova Scotia Health, along with colleagues at Halifax Public Libraries, and it went live in June 2017. Inspired by a presentation on LGBTQ+ health information accessibility at the joint 2016 MLA/CHLA annual conference [48], the librarians collaborated to create a shared guide serving audiences across institutions and the broader community. The process of developing and initial evaluation of the guide was based on the contributions of team members and library interns belonging to the 2SLGBTQIA+ community as well as input from an informal survey of community members, health practitioners, and academic researchers [49]. Beyond the initial survey informing resource inclusion and overall format, the guide has evolved through continued connection and input from prideHealth at Nova Scotia Health [50], web analytics monitoring, and continued connections across academic, hospital, and public libraries.

Since its inception, promotion of the guide has varied: high-visibility links through the public library website, dissemination through organizational newsletters and a regional library association bulletin, and use during individual research consultations [49]. Early promotion efforts also included highlighting the resource at lunch and learn events during local Pride celebrations. Plans are currently underway for a major revamp of the guide based on the results of this study and guide usage statistics, to be accompanied by a collaborative and coordinated outreach campaign.

A highly distinctive characteristic of the Nova Scotia guide is the partnership between the healthcare system, public, and university libraries in the development, maintenance, and promotion of the resource. The potential audience of the guide includes those with multiple relationships to the 2SLGBTQIA+ community (e.g., community members, community health researchers, and healthcare providers). The librarians therefore aimed to provide one unified location for curating resources for various types of users and highlighting resources from all the participating institutions and beyond. However, this collaboration also led to a distinct configuration of the guide, to have it appear in the guide lists of two separate institutional instances of LibGuides. While the entirety of the guide is hosted on the hospital library LibGuide system and features branding from all three contributing institutions, the About page is replicated on the university library system so that it appears in the listing of academic research guides. Other than the duplicated landing page, all other tabs map to the hospital guide to reduce the maintenance workload. The 2SLGBTQIA+ Health Guide has also benefited partnerships beyond the libraries. For example, collaboration with the Nova Scotia Health prideHealth Coordinator has resulted in new projects, most notably the creation and launch of a related guide on Navigating Trans and Gender-diverse Health Care [51].

### Vignette #3: Stony Brook University

The LGBTQ+ Health Research Guide of Stony Brook University Libraries [52] was created in April 2018 by one of the librarians (GS) in the university's Health Sciences Library. This librarian was on the University Libraries' Equality, Inclusion and Diversity Committee during its inaugural year (2017-2018) and he created the guide to support the Libraries' DEI initiatives.

The guide consists of three tabs: (1) the significance of LGBTQ+ health, (2) provider resources, and (3) resources for patients. Half of the consumer health information is focused on resources for locating healthcare, with many Long Island and metro New York City area clinics and services listed. The guide was occasionally featured in a slide show on the library's homepage and was also featured in two library blog posts, including in June 2020 for Pride month. It was not heavily promoted in classes or other orientations, although the librarian who created the guide did introduce it to students who came to the library for consultations on LGBTQ+ topics.

Two years after the research guide was created, Stony Brook Medicine created a separate website with consumer health information [53]. This site was created without the involvement of the library, leading to some overlap in the content. In 2021, a broader Health Disparities research guide was created for the University Libraries [54]. One of that guide's pages reused the provider resources content from the original LGBTQ+ Health guide, as the intended focus of the newer guide was on student and clinician needs.

### Vignette #4: Kansas City University

The LGBTQIA+ Resources Guide [55] was the first of several guides created in 2017 by librarians at Kansas City University to align the library with the university's diversity, equity and inclusion mission. According to the creators, the guide proved to be a critical starting point for hosting LGBTQIA+ informational resources and fostering a safe learning environment on campus. Several LGBTQ+ print and electronic resources have been added to the library collection, and the guide highlights not only library resources, but also local and national consumer health resources. The guide has been featured as a running slide on the library's TV monitors and on the university intranet, as a resource in orientation presentations, and linked to other university pages such as a page for LGBTQ+ Pride Month [56].

### Vignette #5: Virginia Commonwealth University

Two LGBTQIA+ Health research guides at Virginia Commonwealth University (VCU) were launched in September 2019: one addressing provider information needs [57] and the other providing information for community members and patients [58]. A research and education librarian at the Health Sciences Library (EB) created the guides to provide online resources on LGBTQIA+ health for dentistry and dental hygiene students. Each guide aims to direct users to specific and actionable information, such as a directory of providers welcoming to LGBTQIA+ patients, guidance on appropriate terminology, and local resources for social support.

The guide ranges from general LGBTQ+ health information to more focused resources for healthcare providers, such as making a practice welcoming to LGBT clients, patient information guides, and targeted resources on intersex health. The content of each guide was gathered from a variety of sources, including a review of similar guides, the VCU Libraries collection and a list of local organizations and support groups developed by VCU Health's Safe Zone trainers. Preliminary drafts of the guides were reviewed by members of the VCU community with subject matter expertise by training or lived experience, including the consumer health librarian.

The guides were promoted to potential users via a hybrid in-person/online program in September 2019 and again via webinar in January 2020. In addition, information about the guides was presented on the VCU Libraries blog Library Stories in October 2019 [59].

## RESULTS

### Guide Usage Statistics: Clicks on Links

For each of the six guides selected (including the two guides from VCU) we extracted data for the 20 most accessed links; 120 links are thus included in this part of our analysis. As some links are contained in multiple guides, we deduplicated the list based on URLs. After the results were deduplicated, we had 112 unique links in the coding sheet. These links were accessed a total of 2424 times cumulatively during the three-year period.

The most commonly occurring category in the open coding was Community, Local, State, and Provincial Organizations. The resources fitting in this category represented 1292 clicks, or 53.30% of the total clicks. In contrast, the resources for the corresponding category National and International Resources were used 857 times, or 35.35% of the total clicks from the top accessed links. The category, Find a Provider, Service, or Testing Resource, represented links with the second highest frequency of use. Nearly half of the resource clicks (47.65%, or 1155 clicks) were in this category. Together with two similar codes focusing on consumer health, Consumer Health Information (Topic Based) (622 clicks; 25.66%) and Consumer Health “Questions to Ask Your Doctor” Resources (232 clicks; 9.57%), healthcare consumer-directed information constituted a sizable share of the clicks in the sample.

**Figure 1:**
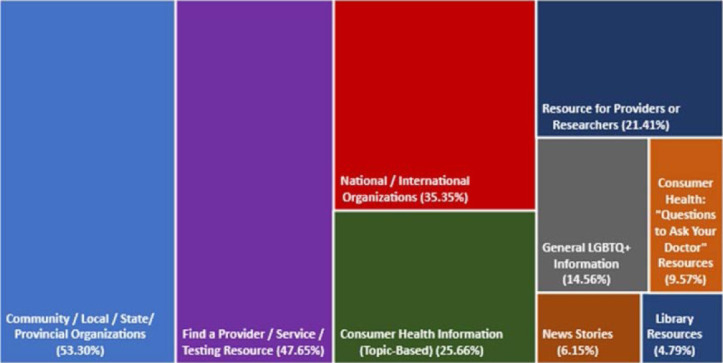
Summary of resource open coding for 112 unique links from top 20 links per guide

Categories centered more on clinical and/or academic information appeared less often in the analysis of the frequently used links. Resources falling under the category Resources for Providers or Researchers only represented 519 clicks (or 21.41%). None of the most-used resources fell under the unused code Resources Specific to School or University, despite the fact that all of the guides were owned or co-owned by academic libraries and had some resources listed that would fit that category. Finally, guide links to library-owned resources such as e-books and journals were rarely used, representing only 116 clicks (4.79%) during the three-year period across all six guides. (See Figure 1 for all codes used and the percentage of user clicks falling under each category.)

### Guide Usage Statistics: Time Series Analyses

Each time series analysis “decomposes” the raw data into three underlying components, which are then plotted for each site. These four subplots for each site are:

1. **(‘y’)** - A plot of the observed (raw) data, i.e. the number of hits for the guide per month
2. **(‘trend’)** - A calculation of the series trend using a moving average. Because the data is monthly (12 observations in one calendar year), the rolling average is calculated using a specific observation, along with the 6 points on either side of it, i.e. 13 points in total. This explains why the first 6 and last 6 observations are missing from the trend plot.
3. **(‘month_avg’)** - The overall cyclical seasonal variation in the data.
4. **(‘irregular’)** - other (often described as ‘random’) influences on the observed data not accounted for by the seasonal variation, for example the influence of one-off promotional events.

### Florida International University

**Figure 2:**
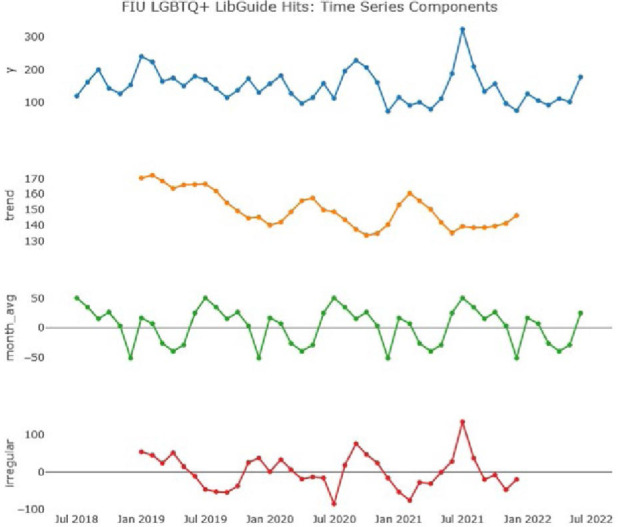
Time series analysis of FIU LibGuide access hits (Aug 2018 - Jun 2022)

For FIU we had access to four complete years of guide hit data: July 2018 to June 2022. The “decomposed” plot for FIU is shown in Figure 2 below.

Our analysis shows a strong seasonal component as well as clear, prominent spikes in the irregular component. This leads us to be confident that we have successfully separated signal from noise in our analysis. Furthermore, the three spikes in the irregular component are very high compared to the monthly average.

The guide shows a gentle downward trend in usage (as shown in the ‘trend’ plot); however, the seasonal variation analysis (‘month avg’) shows two spikes per year; one between July and September, and a smaller one in January. These are unsurprising considering the natural rhythm of activity during the academic year.

The plot of irregular influences on guide usage shows several additional, non-seasonal spikes, separate from any seasonal variations and against the trend. Among most significant are:

- **January 2019**: The cyclical, seasonal plot shows that each January has 15.66 hits above the annual average (shown as ‘0’ in the plot). The extraseasonal, ‘irregular’ result for January 2019 shows a spike of 52.93 hits above this average. The library hosted the National Library of Medicine traveling exhibit Surviving & Thriving: AIDS, Politics, and Culture during this time, when handouts promoting the FIU Transgender Resources Guide were distributed [60].
- **September 2020**: The cyclical, seasonal plot shows that each September has 11.11 hits above the annual average. The extraseasonal, ‘irregular’ result for September 2020 shows a spike of 78.51 hits above this average.

FIU Library participated in the Pride Center Welcome Orientation each September. This iteration had a particularly high attendance due to a combination of the virtual attendance option offered during the pandemic, along with the university's decision to reopen for in-person instruction.

- **July 2021**: The cyclical, seasonal plot shows that each July has 48.24 hits above the annual average. The extraseasonal, ‘irregular’ result for July 2021 shows a large spike of 134.89 hits above this average.

This large uptick in guide usage follows promotional activity during Stonewall Pride in Wilton Manors.

### Nova Scotia Health/Dalhousie University

We conducted separate analyses for two of this LibGuide's four subguides: (1) “About”, and (2) “Community”, as each had high enough usage stats to provide the necessary statistical power.

### Nova Scotia: ‘Community’ Subguide

**Figure 3:**
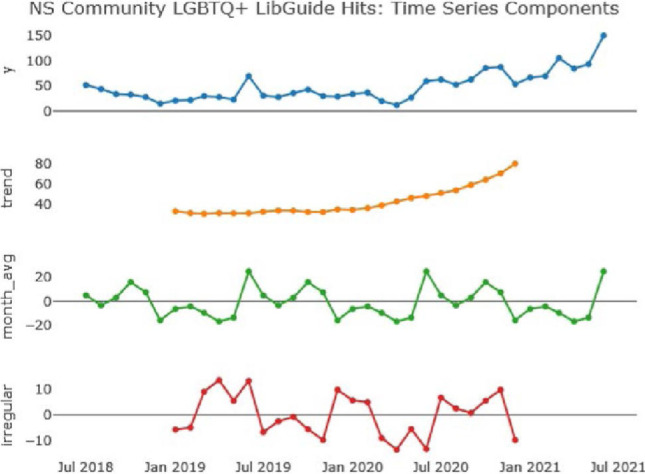
Time series analysis of Nova Scotia ‘Community’ LibGuide access hits (Aug 2018 - Jun 2021)

The decomposed analysis shown in Figure 3 below demonstrates a smooth upward trend in usage (from the ‘trend’ plot). This trend could be attributed to the strong partnership between the hospital library and the Nova Scotia Health prideHealth program, as noted in the vignette, building visibility for the guide through cross-linking and reference in the course of service provision within both units. The analysis also shows an annual usage spike in June and another between September - October (from the ‘month_avg’ plot), reflections on which are provided below.

- **June**: It is likely there are two factors influencing this uptick. Firstly, the guide is promoted each year leading up to Halifax Pride in July. Secondly, access to Dalhousie Library Research Camp modules opens in June. The Guide is the first in the list of LibGuides on the Dalhousie site due to the number at the start (2SLGBTQIA+ Health), therefore any library training including a mention of the Libguide is likely to result in an uptick in clicks to the first page, and possibly beyond.
- **September - October**: Likely due to library promotion of their LibGuides during orientations at Dalhousie University.

The ‘irregular’ variation for this guide at first glance appears to be very significant. However, it is important to note that the scale of variation (from -10 to +10) is much smaller than FIU's, which ranges from -50 to +50. Because of this narrow irregular variation, we believe there are no additional insights to be inferred from this analysis, and that the regular seasonal variation explains nearly all of the spikes in usage of this guide.

### Nova Scotia: “About” Subguide

**Figure 4:**
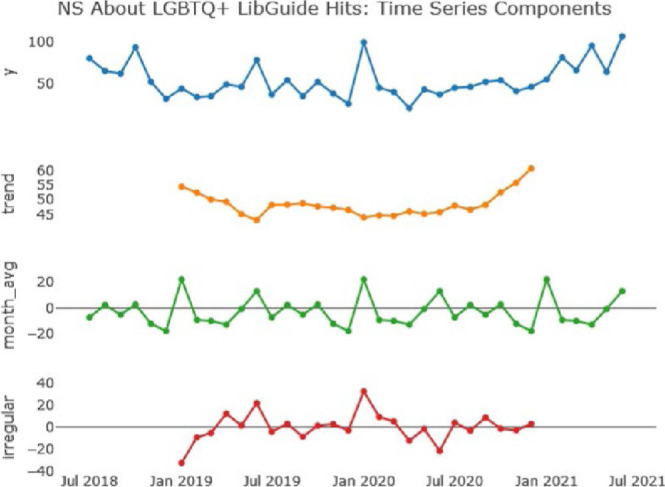
Time series analysis of Nova Scotia ‘About’ LibGuide access hits (Aug 2018 - Jun 2021)

The analysis shown in Figure 4 above has significant similarities with our results for the ‘Community’ subguide, particularly relating to the overall trend, and to the low level of irregular variation in guide usage. There is also a similar annual spike in usage each June, which we believe is likely to be for similar reasons to the same spike in usage for the ‘Community’ guide. There is another spike in January, which we believe is likely due to the annual guide review and maintenance conducted by library staff.

The ‘irregular’ variation for this LibGuide is, similar to the ‘Community’ subguide, very low. We therefore again conclude that it cannot offer any further insights into usage of this guide.

## DISCUSSION

All of the guides in this study are owned or co-owned by academic libraries with health sciences programs whose primary mission is to serve students and faculty with their curricular and research needs. Despite this, the open coding results demonstrate an overwhelming preference for external consumer health resources over clinical/academic information, whether owned by library or publicly available; they are accessing consumer health information potentially in response to their own health concerns or, if these users are health sciences students or health care providers, as clinicians for patients and their families. University students often access health information because of a personal health concern [52], so it is reasonable to conclude that health sciences students may be seeking information or services through their schools' guides [61]. Other academic libraries intentionally provide consumer health information as an outreach tool to both their academic users and to their greater communities [62,63]. Although it is not possible from our analysis to know whether the users of these guides are internal to their academic institutions, or external and unaffiliated, the results indicate that academic health libraries are serving as gateways to vital consumer health information.

We hypothesize that not only are guide users seeking and accessing personal health information, they are also more interested in locally based resources than in nationally based ones (53.3% of clicks for local versus 35.4% for national) and may be seeking resources to help them connect with healthcare; nearly 48 percent of the clicks involved resources with either a provider directory or information on obtaining medical services. This interest in local resources reinforces the recommendation of Stevens and Fajardo that, because they are experts on health information within their own contexts, health sciences librarians can provide value to users by “making it local” when curating the resources in their guides [34]. Even though consumer health resources do not necessarily meet the curricular, research, or clinical needs of their primary constituents, we encourage academic libraries constructing LGBTQ+ health guides to be aware of this strong interest in local resources and to emphasize information of interest to this group, including linking out to corresponding regional LGBTQ+ health organizations.

There are certain times of year when LGBTQ+ health information appears to be more actively sought after by guide users, and these correspond with clear peaks in the seasonality analysis of the time series data. While June, the traditional month for Pride in most of the United States and Canada, is an obvious time for promoting a guide, other days in the LGBTQ+ community calendar are also good options. These include International Transgender Day of Visibility on March 31, National Gay Men's HIV/AIDS Awareness Day on September 27, and Intersex Awareness Day on October 26. The time series analysis for FIU may demonstrate that special events or displays in the library related to the LGBTQ+ community or any of its health disparities could also provide opportunities to promote the guide. FIU's promotion of its transgender health guide on handouts when it hosted the NNLM traveling exhibit, and the resulting uptick in guide usage, is evidence of the effectiveness of this type of outreach activity.

Working with partners outside of the library who share the library's goals can significantly increase both the reach and use of a guide [33,64]. Natural partners for guide promotion within a university or a healthcare organization could include the DEI office or the LGBTQ+ student center. Outside of the institution, additional partners may emerge with effective outreach and networking, as demonstrated by the successful collaboration with the prideHealth program in Nova Scotia. Other possibilities include public libraries, health departments, and LGBTQ+ community organizations. The two guides in this study with the highest traffic both partnered with external agencies for creation and promotion. Much like with the exhibit handout mentioned above, library guides could be promoted in the print or online resources of partner departments, potentially helping both the library and the partner to meet their outreach goals. The FIU guide's increased usage in September 2020, after presenting the guide at their Pride Center's Welcome Orientation, illustrates that traffic can increase after targeted outreach. The time series analyses provide clear evidence of the impact of outreach and promotion work, as can be seen from the peaks in both the seasonal and non-seasonal aspects of the access hits. To more effectively meet the health information needs of students and faculty noted above, targeted outreach and promotional methods are required.

DEI is an increasingly important focus of many health sciences curricula in the United States and in Canada as reflected by recent initiatives to diversify simulated patient cases [65] and integrate topics relevant to minority health [66]. The resources provided in the guides in this study can support these curricular revisions by facilitating discovery and access for students and faculty. While the strong interest in local health resources described above demonstrates the value of academic libraries to the wider public, the relatively low usage of academic and clinical resources raises the question of whether academic LGBTQ+ guides are effectively reaching health sciences students and faculty, both academic and clinical. Direct outreach to faculty and graduate medicine program directors interested in cultural competencies, health equity, and minority health could lead to the inclusion of the guides within program resources and course learning management systems. Library instruction sessions are another possibility; using sample search topics that relate to LGBTQ+ health gives the librarian an opportunity to show the guide to a classroom of students and their instructor. Guides could also be introduced during library orientation sessions with graduate medical students in many residencies. Residents and physicians in many specialties, from family medicine to gynecology, surgery, and endocrinology, need to know where to find LGBTQ+ specific guidelines; LGBTQ+ health guides can therefore play a crucial role in the provision of appropriate patient care.

While some of the guides in our study did receive significant spikes in usage based on outreach and seasonality, not all the guides we looked at had usage high enough to warrant analysis. The LGBTQ+ community is a relatively small segment of the population, comprising approximately 4 to 5 percent of the population in both the United States and Canada [67,68]. Because of the community's low representation in the general population, we consider LGBTQ+ health to be a category of minority health. As such, a guide devoted to it might not receive as much usage as a guide devoted to more mainstream health categories. We would argue that the importance of providing access to quality LGBTQ+ health information should outweigh any concerns of large-scale usage and high impact. These guides support the Healthy People 2030 goals for LGBT health, which include increasing access to HIV medical care and improving the mental and physical well-being of LGBT young people [69]. Creating and maintaining these guides is a professional duty as a health sciences librarian, not only in the traditional librarian role of curating quality health information but also in serving as an advocate for the community and its health and promoting health equity.

There are a couple of limitations to our study. We have made inferences about user preferences and motivations based on our data analysis. However, without communicating directly with users, there is no certainty about their information needs, motivations, or preferences. We have based our interpretations of user motivation on the retrospective analysis of anonymous guide statistics and insights drawn from professional experience working with library users.

By their nature, research guides are subjective, with the librarian actively selecting resources and excludes others based on their professional judgment and expertise. Because of the subjective nature of guides, as well as the limited number of guides in this study, the results are not necessarily indicative of all LGBTQ+ health guides. However, we believe that the insights provided by our analysis will be of interest to all owners of LGBTQ+ health guides.

As a direction for further research, we would suggest updated analyses with a larger number of guides (as these become available) to allow for more robust analysis. We also recommend implementing measures to gauge the impact of the recommendations given above on usage and guide uptake. Another possible direction for further research could be a survey of users. Focus groups or other qualitative research methods could yield deeper insights into user needs and motivations than our analysis was able to do. Focus groups are a widely accepted way of directly incorporating and empowering stakeholders within the research process and can help to verify research findings gleaned from other methodologies [38].

In this study, we have attempted to use retrospective data to assess the use of several existing research guides in meeting the LGBTQ+ health needs of our users. Furthermore, this study serves to provide some insight into how librarians can create new guides and modify existing guides to allow them to have more impact. We have provided a number of suggestions for librarians to improve and promote their guides, most notably with recommendations to focus on local consumer health resources, to engage in targeted marketing and outreach to increase usage, and to partner with other offices or organizations outside of their library. We have also shown a correlation between outreach and guide usage which would not have been clear from a simple visual examination of the raw data. We encourage librarians to conduct their own time series analyses on the usage of their own guides to show the impact of their guides.

The information needs and patterns of library users are often complex. We hope that this study helps to quantify some information seeking behavior relating to LGBTQ+ health, supporting the ideas proposed in the existing literature. More importantly, we hope that more librarians will consider creating LGBTQ+ health guides using the recommendations described above, as a tool to promote health equity and provide users with valid and reliable health information. This guide usage study also cannot shed light on any potential impacts on health services access or health outcomes.

## Data Availability

Data associated with this article are available through the University of Massachusetts Chan Medical School digital repository at https://doi.org/10.13028/ygpj-b407.

## SUPPLEMENTAL FILES

**Appendix A:** R Syntax for TS Analysis

## References

[1] Berliant M, Odorizzi S, Leppard J. Out of the closet and into the waiting room: improving care of 2SLGBTQIA+ patients in the emergency department. Can J Emerg Med. 2021 Nov;23(6):733–6. 10.1007/s43678-021-00202-y 34709585

[2] Kelly-Brown J, Palmer Kelly E, Obeng-Gyasi S, Chen J, Pawlik TM. Intersectionality in cancer care: A systematic review of current research and future directions. Psycho-Oncology. 2022 May;31(5):705–16. 35199401 10.1002/pon.5890

[3] Snyder M. Health Care Experiences of Lesbian Women: A Metasynthesis. Advances in Nursing Science. 2019 Jan;42(1):E1–21. 10.1097/ANS.0000000000000226 30325742

[4] Green KE, Feinstein BA. Substance use in lesbian, gay, and bisexual populations: an update on empirical research and implications for treatment. Psychol Addict Behav. 2012 Jun;26(2):265–78. 22061339 10.1037/a0025424 PMC3288601

[5] King M, Semlyen J, Tai SS, Killaspy H, Osborn D, Popelyuk D, Nazareth I. A systematic review of mental disorder, suicide, and deliberate self harm in lesbian, gay and bisexual people. BMC Psychiatry. 2008 Aug 18;8:70. 18706118 10.1186/1471-244X-8-70 PMC2533652

[6] McCabe SE, West BT, Hughes TL, Boyd CJ. Sexual orientation and substance abuse treatment utilization in the United States: results from a national survey. J Subst Abuse Treat. 2013 Jan;44(1):4–12. 22444421 10.1016/j.jsat.2012.01.007 PMC3388170

[7] Eliason MJ, Sanchez-Vaznaugh EV, Stupplebeen D. Relationships between Sexual Orientation, Weight, and Health in a Population-Based Sample of California Women. Women's Health Issues. 2017 Sep 1;27(5):600–6. 28551076 10.1016/j.whi.2017.04.004 PMC6816305

[8] Pharr JR, Kachen A, Cross C. Health disparities among sexual gender minority women in the United States: A population-based study. J Community Health. 2019 Aug;44(4):721–8. 30830552 10.1007/s10900-019-00631-y

[9] Ramchandani MS, Golden MR. Confronting Rising STIs in the Era of PrEP and Treatment as Prevention. Curr HIV/AIDS Rep. 2019 Jun;16(3):244–56. 31183609 10.1007/s11904-019-00446-5 PMC6582987

[10] Scheim AI, Baker KE, Restar AJ, Sell RL. Health and Health Care Among Transgender Adults in the United States. Annu Rev Public Health. 2022 Apr 5;43(1):503–23. 34882432 10.1146/annurev-publhealth-052620-100313

[11] Tami A, Ferguson T, Bauer GR, Scheim AI. Avoidance of primary healthcare among transgender and non-binary people in Canada during the COVID-19 pandemic. Preventive Medicine Reports. 2022 Jun;27:101789. 35402149 10.1016/j.pmedr.2022.101789 PMC8979617

[12] Augustaitis L, Merrill LA, Gamarel KE, Haimson OL. Online transgender health information seeking: Facilitators, barriers, and future directions. In: Proceedings of the 2021 CHI Conference on Human Factors in Computing Systems [Internet]. Yokohama Japan: ACM; 2021 [cited 2023 Mar 9]. p. 1–14. Available from: https://dl.acm.org/doi/10.1145/3411764.3445091.

[13] GLAAD. Open Letter to Facebook [Internet]. GLAAD. 2019 [cited 2023 Mar 8]. Available from: https://www.glaad.org/blog/open-letter-facebook

[14] Lapinski J, Diaz KM. Single Accreditation System for Graduate Medical Education: An Opportunity for Lesbian, Gay, Bisexual, and Transgender Health Education Integration in Osteopathic Medicine. 2016;116(2):3. 10.7556/jaoa.2016.018 26830521

[15] Ploumen R, Livas C. Students' awareness of LGBT resources in Dutch dental schools. J Dent Educ. 2020;84(8):881–6. 32086818 10.1002/jdd.12112

[16] Tartavoulle T, Landry J. Educating Nursing Students About Delivering Culturally Sensitive Care to Lesbian, Gay, Bisexual, Transgender, Questioning/Queer, Intersex, Plus Patients: The Impact of an Advocacy Program on Knowledge and Attitudes. Nurs Educ Perspect. 2021 Aug;42(4):E15. 33935246 10.1097/01.NEP.0000000000000819

[17] Zumwalt AC, Carter EE, Gell-Levey IM, Mulkey N, Streed CG, Siegel J. A Novel Curriculum Assessment Tool, Based on AAMC Competencies, to Improve Medical Education About Sexual and Gender Minority Populations. Academic Medicine. 2022 Apr;97(4):524–8. 34108379 10.1097/ACM.0000000000004203

[18] Waryold JM, Kornahrens A. Decreasing barriers to sexual health in the lesbian, gay, bisexual, transgender, and queer community. Nurs Clin North Am. 2020 Sep;55(3):393–402. 32762858 10.1016/j.cnur.2020.06.003

[19] Fikar CR, Keith L. Information needs of gay, lesbian, bisexual, and transgendered health care professionals: results of an Internet survey. J Med Libr Assoc. 2004 Jan;92(1):56–65. 14762463 PMC314103

[20] Morris M, Roberto KR. Information-seeking behaviour and information needs of LGBTQ health professionals: a follow-up study. Health Info Libr J. 2016 Sep;33(3):204–21. 27060995 10.1111/hir.12139

[21] McLeod DW, Miller AV. Medical, social & political aspects of the acquired immune deficiency syndrome (AIDS) crisis: a bibliography [Internet]. Canadian Gay Archives; 1985 [cited 2023 May 26]. Available from: https://tspace.library.utoronto.ca/handle/1807/17361.

[22] Malinowsky HR, Perry GJ. AIDS information sourcebook. Phoenix: Oryx Press; 1991.

[23] Huber JT. How to find information about AIDS. 2nd ed. New York: Haworth Press; 1992.

[24] Perry GJ. The activist health sciences librarian. jmla [Internet]. 2020 Jan 2 [cited 2023 Feb 9];108(1). Available from: http://jmla.pitt.edu/ojs/jmla/article/view/859. 10.5195/jmla.2020.859 PMC6920003 31897047

[25] Comulada WS, Step M, Fletcher JB, Tanner AE, Dowshen NL, Arayasirikul S, Keglovitz Baker K, Zuniga J, Swendeman D, Medich M, Kao UH, Northrup A, Nieto O, Brooks RA, Special Projects Of National Significance Social Media Initiative Study Group. Predictors of Internet Health Information–Seeking Behaviors Among Young Adults Living With HIV Across the United States: Longitudinal Observational Study. J Med Internet Res. 2020 Nov 2;22(11):e18309. 33136057 10.2196/18309 PMC7669436

[26] Rose ID, Friedman DB. We need health information too: A systematic review of studies examining the health information seeking and communication practices of sexual minority youth. Health Educ J. 2013 Jul;72(4):417–30.

[27] Walsh JL, Zarwell M, John SA, Quinn KG. Sources of Information about Pre-Exposure Prophylaxis (PrEP) and Associations with PrEP Stigma, Intentions, Provider Discussions, and Use in the United States. J Sex Res. 2022 Aug 29;1–13. 10.1080/00224499.2022.2110208 PMC9971350 36036718

[28] Pho AT, Bakken S, Lunn MR, Lubensky ME, Flentje A, Dastur Z, Obedin-Maliver J. Online health information seeking, health literacy, and human papillomavirus vaccination among transgender and gender-diverse people. J Am Med Inform Assoc. 2022 Jan 12;29(2):285–95. 34383916 10.1093/jamia/ocab150 PMC8757308

[29] Tenney C, Surkan KJ, Hammond Gerido L, Betts-Green D. Crisis of Erasure: Transgender and Gender-Nonconforming Populations Navigating Breast Cancer Health Information. IJIDI. 2021 Dec 21;5(4):132–49.

[30] Drake AA, Bielefield A. Equitable access: Information seeking behavior, information needs, and necessary library accommodations for transgender patrons. Library & Information Science Research. 2017 Jul;39(3):160–8.

[31] Hawkins BW, Morris M, Nguyen T, Siegel J, Vardell E. Advancing the conversation: next steps for lesbian, gay, bisexual, trans, and queer (LGBTQ) health sciences librarianship. J Med Libr Assoc. 2017 Oct;105(4):316–27. 28983195 10.5195/jmla.2017.206 PMC5624421

[32] Morris M, Hawkins B. Towards a new specialization in health librarianship: LGBTQ health. J Can Health Libr Assoc. 2016 Apr 1;37(1):20–3.

[33] Stevens GA, Morris M, Nguyen T, Vardell E. Health sciences librarians in the field: Advocating and campaigning for LGBTQ+ health. In: Mehra B, editor. LGBTQ+ Librarianship in the 21st Century: Emerging Directions of Advocacy and Community Engagement in Diverse Information Environments [Internet]. Bingley, United Kingdom: Emerald Group Publishing; 2019. p. 65–87. (Advances in Librarianship; vol. 45). Available from: 10.1108/S0065-283020190000045015.

[34] Stevens GA, Fajardo FJ. LGBTQ+ health research guides at North American health sciences libraries: a survey and content analysis. jmla. 2021 Oct 5;109(3):406–13. 34629969 10.5195/jmla.2021.1189 PMC8485968

[35] Ouellette D. Subject Guides in Academic Libraries: A User-Centred Study of Uses and Perceptions. Canadian Journal of Information and Library Science. 2011;35(4):436–51.

[36] Kouame G, Hendren S. Library tools at the nurses' station: exploring information-seeking behaviors and needs of nurses in a war veterans nursing home. J Med Libr Assoc. 2022 Apr 26;110(2):159–65. 35440895 10.5195/jmla.2022.1357 PMC9014917

[37] Emmel N. Purposeful Sampling. In: Sampling and Choosing Cases in Qualitative Research: A Realist Approach [Internet]. 1 Oliver's Yard, 55 City Road, London EC1Y 1SP United Kingdom: SAGE Publications Ltd; 2013 [cited 2023 May 22]. Available from: https://methods.sagepub.com/book/sampling-and-choosing-cases-in-qualitative-research.

[38] Robson C. Real world research: a resource for social scientists and practitioner-researchers. 2nd ed. Oxford, UK: Blackwell Publishers; 2002. 599 p.

[39] Clairoux N, Morris M, Brown HL. Academic Dental Librarianship in Canada: Taking Stock, Planning the Future. J Can Health Libr Assoc. 2017 Dec 1;38(3):102–17.

[40] R Foundation. R: The R Project for Statistical Computing [Internet]. [cited 2022 Nov 9]. Available from: https://www.r-project.org/.

[41] RStudio Team. RStudio: Integrated Development for R [Internet]. Boston, MA: RStudio, Inc.; 2018. Available from: http://www.rstudio.com/.

[42] Krispin R. TSstudio: Functions for Time Series Analysis and Forecasting [Internet]. 2020 [cited 2022 Nov 9]. Available from: https://CRAN.R-project.org/package=TSstudio.

[43] Krispin R. Decomposition of Time Series [Internet]. 2020 [cited 2022 Nov 9]. Available from: https://ramikrispin.github.io/halloween-time-series-workshop/ts-decomposition.html.

[44] Shumway RH, Stoffer DS. Time Series Analysis and Its Applications: With R Examples. 4th ed. Cham: Springer International Publishing; 2017. 1 p. (Springer Texts in Statistics).

[45] Fajardo F. Transgender Resources [Internet]. 2022 [cited 2022 Dec 2]. Available from: https://libguides.medlib.fiu.edu/c.php?g=337583&p=2273717.

[46] Holder CL, Perez-Gilbe HR, Fajardo FJ, Garcia S, Cyrus E. Disparities of HIV risk and PrEP use among transgender women of color in South Florida. J Natl Med Assoc. 2019 Dec;111(6):625–32. 31526532 10.1016/j.jnma.2019.08.001

[47] McLean K, Parker R. 2SLGBTQIA+ Health: About [Internet]. 2022 [cited 2022 Dec 2]. Available from: https://library.nshealth.ca/LGBTQ/About.

[48] Hawkins B, Dyck DR, Morris M, Nguyen T, Siegel J, Vardell E. Creating a Needed Dialogue: A Discussion about Lesbian, Gay, Bisexual, Transgender, Questioning (LGBTQ) Health Librarianship in 2016. Mosaic ’16: Joint Meeting of the Medical Library Association (MLA), the Canadian Health Libraries Association / Association des bibliothèques de la santé du Canada (CHLA/ABSC), and the International Clinical Librarian Conference (ICLC); 2016 May 16; Toronto, ON.

[49] Parker R, McLean K, Sternberg S. Partnerships for Better Health Information: The LGBTQ+ Health Guide from Dalhousie Libraries, Nova Scotia Health Authority Library Services, and Halifax Public Libraries. APLA Bulletin [Internet]. 2017 Nov 3;81(2). Available from: https://ojs.acadiau.ca/index.php/aplabulletin/article/view/1814.

[50] Nova Scotia Health Authority. prideHealth [Internet]. 2022 [cited 2022 Dec 2]. Available from: https://www.nshealth.ca/content/pridehealth.

[51] McLean K. Navigating Trans and Gender-diverse Health Care: Home [Internet]. 2022 [cited 2022 Dec 2]. Available from: https://library.nshealth.ca/TransGenderDiverse/Home.

[52] Saragossi J. LGBTQ+ Health [Internet]. 2022 [cited 2022 Dec 2]. Available from: https://guides.library.stonybrook.edu/c.php?g=827067&p=5904865.

[53] Stony Brook Medicine. LGBTQ* Care at Stony Brook Medicine [Internet]. 2022 [cited 2022 Dec 2]. Available from: https://www.stonybrookmedicine.edu/LGBTQ.

[54] Saragossi J. Health Disparities and Equity in Health Care [Internet]. 2022 [cited 2022 Dec 2]. Available from: https://guides.library.stonybrook.edu/c.php?g=1096086&p=7993427.

[55] Fitterling L. LGBTQIA+ Health Resources [Internet]. 2022 [cited 2022 Dec 2]. Available from: https://kansascity.libguides.com/c.php?g=790749&p=5657902.

[56] Fitterling L. Personal Communication. 2022.

[57] Brody E. LGBTQIA+ Health [Internet]. 2022 [cited 2022 Dec 2]. Available from: https://guides.library.vcu.edu/c.php?g=960434&p=6934606.

[58] Ladd D. Lesbian, Gay, Bisexual, Transgender, and Intersex Health: LGBTQIA+ Healthcare and Social Support [Internet]. 2022 [cited 2022 Dec 2]. Available from: https://guides.library.vcu.edu/c.php?g=935962&p=6745970.

[59] VCU Libraries. VCU Libraries launches guides for LGBTQIA+ Health [Internet]. 2019 [cited 2022 Sep 12]. Available from: https://blogs.vcu.edu/librarystories/2019/10/17/vcu-libraries-launches-guides-for-lgbtqia-health/.

[60] National Library of Medicine. Making Exhibition Connections: Florida International University Herbert Wertheim College of Medicine [Internet]. Circulating Now from NLM. 2020 [cited 2022 Sep 28]. Available from: https://circulatingnow.nlm.nih.gov/2020/03/03/making-exhibition-connections-florida-international-university-herbert-wertheim-college-of-medicine/.

[61] Duhon L, Jameson J. Health information outreach: a survey of U.S. academic libraries, highlighting a midwestern university's experience. Health Info Libr J. 2013 Jun;30(2):121–37. 23692453 10.1111/hir.12017

[62] Kirkpatrick N, Dixson MA. An Academic Library Utilization of Research Guides to Disseminate Consumer Health Resources. J Consum Health Internet. 2020 Oct 1;24(4):430–8.

[63] Tringali B. Health Promotion, Collaboration, and Outreach: Creating Space for Health Literacy at a Specialized, Academic Research Library. JLOE. 2021 Sep 13;1(2):14–21.

[64] Goodsett M, Dougan K. Community Outreach Through LibGuides. In: Tyckoson DA, Dove JG, editors. Reimagining Reference in the 21st Century [Internet]. Purdue University Press; 2014 [cited 2022 Nov 30]. Available from: http://www.jstor.org/stable/10.2307/j.ctt6wq3n2.

[65] Conigliaro RL, Peterson KD, Stratton TD. Lack of Diversity in Simulation Technology: An Educational Limitation? Sim Healthcare. 2020 Apr;15(2):112–4. 10.1097/SIH.0000000000000405 32044854

[66] Pregnall AM, Churchwell AL, Ehrenfeld JM. A Call for LGBTQ Content in Graduate Medical Education Program Requirements. Academic Medicine. 2021 Jun;96(6):828–35. 34031304 10.1097/ACM.0000000000003581

[67] Statistics Canada. The Daily — A statistical portrait of Canada's diverse LGBTQ2+ communities [Internet]. 2021 [cited 2022 Aug 26]. Available from: https://www150.statcan.gc.ca/n1/daily-quotidien/210615/dq210615a-eng.htm.

[68] Williams Institute, UCLA School of Law. LGBT Data & Demographics [Internet]. 2019 [cited 2020 Aug 28]. Available from: https://williamsinstitute.law.ucla.edu/visualization/lgbtstats/?topic=LGBT.

[69] Office of Disease Prevention and Health Promotion. LGBT [Internet]. Healthy People 2030. [cited 2022 Nov 30]. Available from: https://health.gov/healthypeople/objectives-and-data/browse-objectives/lgbt.

