# Supplementary material for: LGBTQ+ health research guides: a multi-institutional analysis of usage patterns and user information needs
Source: J Med Libr Assoc. 2023 Oct 2;111(4):762–73. doi: 10.5195/jmla.2023.1661 (PMC10621682; doi:10.5195/jmla.2023.1661)
Supplement: Supplementary file 1 — Appendix A: R Syntax for TS Analysis [file jmla-111-4-762-s01.pdf]

Appendix A: R syntax for TS analysis

```
library(tidyverse)
library(plotly)
library(lubridate)
library(TSstudio)

# load data for site (here FIU as example)
fiu <- read_csv('R/<filename.csv>, col_names=F)
fiu_ts <- ts(fiu, frequency=12, start=c(2018,7))

# calculating trend component
fiu_smooth <- ts_ma(fiu_ts, n=6, separate=F)

# removing trend component from raw time series
df <- ts_to_prophet(fiu_ts) %>%
      select(date = ds, y) %>%
      left_join(ts_to_prophet(fiu_smooth$ma_6) %>%
      select(date = ds, trend = y), by = "date")

df$detrend <- df$y - df$trend

# calculating seasonal component
df$year <- year(df$date)
df$month <- month(df$date)

fiu_seasonal <- df %>%
      group_by(month) %>%
      summarise(month_avg = mean(detrend, na.rm = T), .groups = "drop")

# calculating irregular component
df <- df %>% left_join(fiu_seasonal, by = "month")
df$irregular <- df$y - df$trend - df$month_avg

# creating final plot
ts_plot(df[, c("date", "y", "trend", "month_avg", "irregular")],
      title = "FIU LGBTQ+ LibGuide Hits: Time Series Components",
      type="multiple", line.mode = "lines+markers")
```
